# Strong evidence of mitochondrial polyphyly of the *Leopardus tigrinus* (Mammalia, Felidae) species complex revealed by expanded analyses of Andean populations

**DOI:** 10.1590/1678-4685-GMB-2025-0231

**Published:** 2026-07-17

**Authors:** Diana Lucía Buitrago-Torres, Alejandra Bonilla-Sánchez, Isadora Teresa França Pereira, Caroline Charão Sartor, Tatiane Campos Trigo, Giovanna Medeiros Tavares de Oliveira, Tadeu Gomes de Oliveira, Fernando Angulo, Lizette Bermudez Larrazábal, Gianmarco Rojas Moreno, Jorge L. Ramirez, Mariano Mavila, Tatiana Quevedo, Héctor E. Ramírez-Chaves, Paola Pulido-Santacruz, Larissa R. Oliveira, Eduardo Eizirik

**Affiliations:** 1 Pontifícia Universidade Católica do Rio Grande do Sul (PUCRS), Escola de Ciências da Saúde e da Vida, Centro de Biologia Genômica e Molecular, Porto Alegre, RS, Brazil.; 2 Universidad de Antioquia, Instituto de Biología. Facultad de Ciencias Exactas y Naturales, Grupo Mastozoología y Colección Teriológica, Medellín, Antioquia, Colombia.; 3 Universidade Vila Velha, Vila Velha, ES, Brazil.; 4 University of Oxford, Wildlife Conservation Research Unit, Department of Biology, Oxford, UK.; 5 Secretaria do Meio Ambiente e Infraestrutura, Museu de Ciências Naturais do Rio Grande do Sul, Setor de Mastozoologia, Porto Alegre, Rio Grande do Sul, Brazil.; 6 Universidade Estadual do Maranhão, Departamento de Biologia, São Luís, MA, Brazil.; 7 Instituto Pró-Carnívoros, Atibaia, SP, Brazil.; 8 IUCN, Species Survival Commission, Cat Specialist Group, Ittigen, Switzerland; 9 Centro de Ornitología y Biodiversidad (CORBIDI), Peru.; 10 Navi Fauna, Peru.; 11 Universidad Ricardo Palma, Facultad de Ciencias Biológicas, Escuela de Medicina Veterinaria, Peru.; 12 Universidad Nacional Mayor de San Marcos, Facultad de Ciencias Biológicas, Lima, Peru.; 13 Parque Sinchi Roca, Comas, Lima, Peru.; 14 Parque Huascar, Villa El Salvador, Lima, Peru.; 15 Zoológico Patronato Parque de Las Leyendas, Lima, Peru.; 16 Universidad de Caldas, Facultad de Ciencias Exactas y Naturales, Departamento de Ciencias Biológicas, Grupo de Investigación Genética, Biodiversidad y Manejo de Ecosistemas (GEBIOME), Manizales, Caldas, Colombia.; 17 Universidad de Caldas, Centro de Museos, Museo de Historia Natural, Manizales, Caldas, Colombia.; 18 Universidad del Rosario, Escuela de Ciencias e Ingeniería, Bogotá, Colombia.; 19 Universidade do Vale do Rio dos Sinos (UNISINOS), Laboratório de Ecologia de Mamíferos, São Leopoldo, Brazil.; 20 Grupo de Estudos de Mamíferos Aquáticos do Rio Grande do Sul (GEMARS), Torres, RS, Brazil; 21 Associação de Pesquisa e Preservação de Ecossistemas Aquáticos (AQUASIS), Caucaia, CE, Brazil; 22 Universidade Federal do Ceará, Programa de Pós-Graduação em Engenharia de Pesca, Marine Vertebrate Evolution and Conservation Lab, Fortaleza, CE, Brazil; 23 Universidade Federal do Rio Grande do Sul, Programa de Biologia Animal, Porto Alegre, RS, Brazil.

**Keywords:** Mammalia, Neotropics, mitochondrial DNA, Phylogenetics

## Abstract

Over the past two decades, molecular data have revealed a complex evolutionary history for the Neotropical felid genus *Leopardus*. A particularly problematic subset has been the *L. tigrinus* complex, which has been demonstrated to comprise more than one species. Recent molecular data indicated that it is not even monophyletic, comprising distinct *Leopardus* lineages with a similar morphology, which likely underlies the assumption that they formed a single species. To further investigate its composition and evolutionary history, we generated mtDNA data from multiple individuals sampled in Andean regions of Colombia and Peru. The Colombian samples formed a well-supported clade that was the sister-group of the Costa Rican lineage. Remarkably, the Peruvian *L. tigrinus* samples formed a different clade, placed at a distinct location within the *Leopardus* phylogeny, as a sister-group to (*L. pardalis* + *L. wiedii*). This unexpected result extends the inference of non-monophyly of the *L. tigrinus* complex, and raises the possibility that additional taxonomic entities may be contained in this genus.

*Leopardus* (Gray, 1982) is the most speciose genus of the Felidae, comprising between eight and 13 recognized species. It consists of an endemic Neotropical radiation that diverged from other felid lineages *ca.* 10 million years ago (MYA) and began its own diversification *ca.* 3 - 4.5 MYA (Li et al., 2016; Trindade et al., 2021; Lescroart et al., 2023). The genus has been the focus of several phylogenetic and taxonomic studies, which have revealed that it has had a much more complex evolutionary history than previously envisioned (e.g., García-Perea, 1994; Johnson et al., 1999; Trigo et al., 2008, 2013; Nascimento and Feijó, 2017; Nascimento *et al.,* 2021; Trindade *et al.,* 2021; Lescroart *et al.,* 2023). These studies provided evidence that *L. tigrinus* (commonly known as ‘tigrinas’ or ‘tiger cats’) and *L. colocola* are actually two species complexes, indicating that the taxonomic diversity of *Leopardus* may be considerably underestimated. In addition, genetic data (Trigo *et al.,* 2008, 2013; Santos et al., 2018) revealed massive introgression of *L. colocola* (or specifically its subset *L. braccatus*, following the taxonomic proposition of Nascimento *et al.* (2021)) mitochondrial DNA (mtDNA) into the central and northeastern Brazilian populations of *L. tigrinus* (specifically referred to as *L. emiliae* in the taxonomic proposition of Nascimento and Feijó (2017)). Furthermore, these genetic studies have revealed a current, secondary hybridization process between *L. guttulus* (the southernmost component of the ‘*L. tigrinus*’ complex (see below)) and another congener, *L. geoffroyi*, adding another layer of complexity to the evolution of this genus (Trigo *et al.,* 2008, 2013; Sartor et al., 2021).

Prior to the realization that *L. tigrinus* comprises a complex, it was treated as a single, widely distributed species, occurring from Costa Rica to northern Argentina, and comprising four subspecies (e.g., Johnson et al., 1999; Wozencraft 2005): *L. t. oncilla* in Costa Rica and Panama, *L. t. pardinoides* in northwestern South America, *L. t. tigrinus* in the Guiana shield and north/northeastern Brazil, and *L. t. guttulus* in southern Brazil, Paraguay, and northeastern Argentina. An initial indication that it might comprise more than one evolutionary lineage was presented by Johnson *et al.* (1999), who found a deep divergence between *L. t. oncilla* and *L. t. guttulus* mtDNA sequences. This finding was corroborated by Trigo et al. (2008), also with mtDNA data, and demonstrated conclusively by Trindade et al. (2021) and Lescroart et al. (2023) with genome-wide data. In addition, Trigo *et al.* (2013) revealed that there was no gene flow between *L. tigrinus* populations sampled in northeastern (NE) and south-southeastern (SSE) Brazil, leading to the recognition of the latter as a distinct species, *L. guttulus*. This finding was supported by morphological analyses (Nascimento and Feijó, 2017) and formally recognized by the most recent felid taxonomic reference source (Kitchener et al., 2017).

The morphological analyses of Nascimento and Feijó (2017) indicated that *L. tigrinus* should be divided into two species: *L. tigrinus* in Central America, Andean region and Guiana Shield; and *L. emiliae* in NE Brazil. The reference work by Kitchener et al. (2017) provisionally maintained *L. tigrinus* as a single species, recognizing two subspecies (*L. t. tigrinus* in South America and *L. t. oncilla* in Central America) and indicating that additional units, such as *L. t. pardinoides* in the Andean region, may warrant species-level recognition pending additional research. Subsequently, Ruiz-García et al. (2018, 2023) suggested that Andean *L. tigrinus* populations harbor several mitochondrial haplogroups, including a single individual from southern Colombia that they hypothesized to represent a distinct species due to its phylogenetic distinctiveness. Due to the use of different mtDNA gene segments, those results could not be directly compared to previous studies focusing on the mitochondrial phylogeny of the *L. tigrinus* complex (e.g., Trigo et al., 2008, 2013). Moreover, since the nuclear datasets reported by Trigo *et al.* (2013) and Trindade et al. (2021) did not include Andean samples, the phylogenetic relationships of these *L. tigrinus* populations could not be addressed in those studies. A recent analysis of whole-genome sequences has included two Andean *L. tigrinus* individuals from Colombia, and showed them to be the sister-group of the Central American sample, and distinct from other South American lineages (Lescroart et al., 2023). An extensive analysis that included morphological, biogeographic and ecological niche information (de Oliveira et al., 2024), supported by the genomic findings of Lescroart *et al.* (2023), revealed three distinct species: *L. pardinoides* (that was raised to species level and included the former *L. p. oncilla* as a subspecies) from the cloud forests of Central American and Andean Cordilleras, *L. tigrinus* from the savannas of the Guiana Shield and central/northeastern Brazil, and *L. guttulus* from the Atlantic Forest domain. These intriguing results highlight the need to survey additional Andean individuals, including populations from different areas, to assess their evolutionary relationships and clarify their taxonomic status. An initial step in this survey is to investigate these groups with mtDNA markers that can be directly compared with ascertained data from other regions, so as to characterize their matrilineal evolutionary history and help direct further genomic studies.

To achieve this goal, we generated DNA sequences from multiple Colombian and Peruvian *L. tigrinus* complex individuals, targeting the same fragment of the mitochondrial gene *ND5* that had been used previously to characterize this species complex (Trigo et al., 2008, 2013). We analyzed a mtDNA fragment containing a portion of the *ND5* gene (positions 12521-13087 of the *L. tigrinus* sequence (NCBI: NC_028317) reported by Li et al. (2016)) from 53 individuals of genus *Leopardus*, along with three outgroup species (*Caracal caracal, Lynx lynx* and *Puma concolor*; NCBI accession numbers KP202272.1, KP202283.1 and KP202261.1, respectively). Most of the *Leopardus* samples were sequenced for this study from blood or tissue samples housed in the collection of the PUCRS Laboratory of Genomics and Molecular Biology (Brazil). Six samples from Colombia were obtained from museum-preserved specimens deposited in the Mammal collection of the *Instituto Alexander von Humboldt* (IAvH-M) or from fresh tissues deposited in the Humboldt’s tissue collection (IAvH-CT). Eight Peruvian individuals were represented by blood samples obtained from captive animals hosted in Huachipa Zoo, Parque Huáscar, Parque Sinchi Roca and Patronato Parque de las Leyendas Zoo (see Table S1 in Supplementary Information for sample details).

DNA extractions were performed either with the QIAamp^®^ DNA Blood Mini Kit (Qiagen), with a standard phenol-chloroform protocol (Sambrook and Russell, 2006), or with a combination of both methods (for museum samples). We amplified the target fragment using the primers ND5-DF1 and ND5-DR1 (Trigo et al., 2008), as well as novel internal primers (Table S2) designed for *Leopardus* species, targeting short sub-fragments to maximize PCR efficiency with degraded samples, such as museum pelts. Each mtDNA fragment was amplified individually using the PCR protocols described by Trigo *et al.* (2008, 2013) and subsequently sequenced using Sanger technology.

Sequences generated here were complemented by others that had been previously reported by our group (Trigo et al., 2008, 2013), as well as the homologous segment of the *L. jacobita* mtDNA extracted from the mitogenome reported by Li et al. (2016) and downloaded from NCBI (NC_028322.1). This led to a nucleotide matrix comprising 563 bp and 55 individuals comprising all currently recognized *Leopardus* species and multiple populations belonging to the *L. tigrinus* complex, including Andean samples from Colombia and Peru. Specifically, the dataset comprised 24 individuals identified as part of the *L. tigrinus* complex (including *L. guttulus* and *L. pardinoides*), as well as representatives of the *L. colocola* complex (n=5), *L. geoffroyi* (n=5), *L. guigna* (n=2), *L. jacobita* (n=1), *L. pardalis* (n=8) and *L. wiedii* (n=8), along with the three individuals used as outgroups. We aligned this dataset using the MUSCLE algorithm within MEGA X (Kumar et al., 2018).

We reconstructed phylogenetic trees from the resulting alignment by applying two optimality criteria: a Maximum Likelihood (ML) approach in IQ-TREE v2 (Minh et al., 2020), and a Bayesian Inference (BI) approach in BEAST2 (Bouckaert et al., 2014). We used the IQ-TREE ModelFinder tool (Kalyaanamoorthy et al., 2017) to identify the best-fit nucleotide substitution model for our dataset, which was HKY+F+G4 (BIC=5523.465; see Table S3). IQ-TREE uses a stochastic algorithm for finding initial ML trees. The ML tree generated with the highest log likelihood is presented in this study. Additionally, 10,000 ultrafast Bootstrap (UFboot) (Hoang et al., 2018) replications, as implemented in IQ-TREE, were performed to assess nodal support. For the Bayesian analysis, we performed three independent runs using the HKY+F+G4 substitution model, a relaxed molecular clock, and a Calibrated Yule Model of speciation. We performed the age calibration with a uniform prior distribution for nodes using the minimum and maximum values of the most recent common ancestor time (MRCA) of the subfamily Felinae (7.42 - 15.48 Mya) and genus *Leopardus* (1.64 -5.03 Mya) reported by Li *et al.* (2016). Although first-order (e.g., fossil) calibrations would be preferred, due to the lack of *Leopardus* fossils with sufficient age and reliable phylogenetic placement, second-order (i.e., molecular) constraints are the only option presently available for this group, and have been regularly employed in our studies (e.g., Lescroart et al., 2023). All Markov chain Monte Carlo (MCMC) runs comprised 50,000 iterations, sampling every 1,000 steps, with a burn-in of 10%. Convergence of the chains was evaluated using Tracer v 1.7 (Rambaut et al., 2018) by assessing the effective sample sizes for all relevant parameters. We combined the results of the three runs in LogCombiner, and summarized them in a Maximum Clade Credibility tree, based on median heights, generated with TreeAnnotator. We then visualized and assessed the convergence of parameters across the iterations with Tracer v 1.7.

In addition to the phylogenetic analyses, we also inferred a haplotype network from our dataset to explore genealogical relationships among mtDNA sequences without assuming a strictly bifurcating evolutionary history. For that, we employed the median-joining algorithm (Bandelt et al., 1999) as implemented in POPART v.1.7 (Leigh and Bryant, 2015).

Both maximum likelihood (ML) and Bayesian Inference (BI) phylogenetic trees yielded high support (ML bootstrap >94% and posterior probability >0.95) for the monophyly of each of the *L. tigrinus* geographic units represented by more than one individual and bearing its own mtDNA lineage (Figure 1 and Figures S1 and S2). In contrast, as observed in previous studies (e.g., Trigo et al., 2013; Santos et al., 2018), the northeastern Brazilian tigrina individuals (NE tigrina) contained mtDNA haplotypes derived from an ancient introgression from the *L. colocola* complex. Therefore, its monophyly and relationships cannot be assessed with mitochondrial data, but both aspects have already been ascertained using genome-wide nuclear markers (Trindade et al., 2021; Lescroart et al., 2023).

**Figure 1 -:**
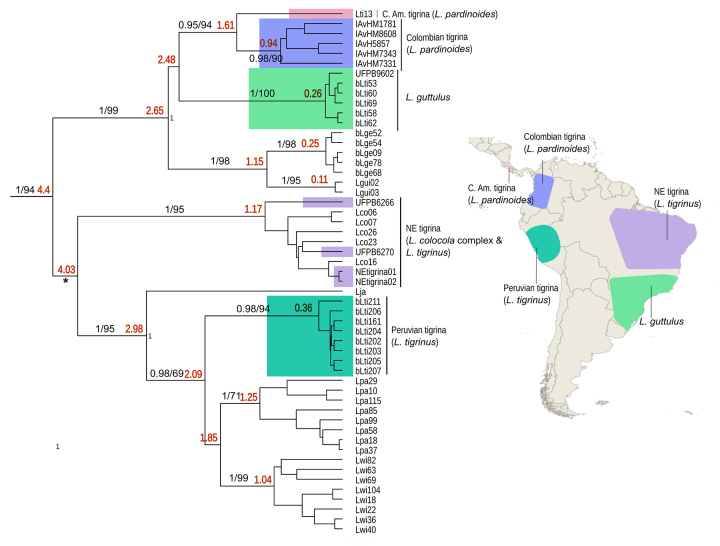
Bayesian maximum clade credibility tree of mtDNA sequences from Neotropical felids of genus *Leopardus*, including all currently sampled geographic units of the *L. tigrinus* complex (color-coded on the map). A congruent tree was obtained with Maximum Likelihood (ML), with a topological difference observed at only one basal node, marked here with an asterisk (see Figure S2). Numbers above the branches indicate support for the adjacent node (Bayesian posterior probability/percent ML bootstrap). Dark red numbers at nodes indicate the age (time to the most recent common ancestor (TMRCA)) of that mtDNA divergence event, in million years ago (Mya) (see Figure S1 for credibility intervals). Support values and divergence dates are displayed only for species-level nodes, higher-level clades and nodes defining additional tigrina units. Sequences belonging to tigrina units are color-coded according to the map.

The monophyly of each tigrina geographic unit is particularly relevant in the case of the Colombian samples, whose clade support was high (posterior probability: 0.98; ML bootstrap: 90%) and whose mitochondrial Time to the Most Recent Common Ancestor (TMRCA) was quite recent (95% HPD: 0.42-1.51 Mya). Since our sample set included the individual (IAvH5857) that had been proposed to represent a distinct species (Ruiz-García et al., 2023), our results do not support such mtDNA phylogenetic uniqueness, and instead indicate that the Colombian tigrinas sampled so far comprise a single evolutionary unit. The finding that sample IAvH5857 belongs within the Colombian lineage (*i.e.*, *L. pardinoides*) corroborates the results from Astorquiza et al. (2023) and Marín-Puerta et al. (2025), which had already indicated this relationship based on sequence-level identity between this specimen and other individuals from that region.

The mitochondrial relationships among the *L. tigrinus* units and the other *Leopardus* species were consistently reconstructed with the ML and BI analyses (see Figure 1 and Figures S1 and S2). Across the whole inferred tree, only one node was incongruent between the BI and ML phylogenies, namely that relating the three main *Leopardus* lineages, now recognized as subgenera (Lescroart et al., 2023; see below for additional details). The north Andean (*i.e.*, Colombian) clade was retrieved as the sister-group of the Central American (C. Am.) sample (BI posterior probability 0.95; ML bootstrap 94%), consistent with the mitochondrial and nuclear results reported by Lescroart *et al.* (2023). Our estimate of the mtDNA TMRCA between Colombian and C. Am. units was *ca.* 1.6 Mya, considerably older than the coalescent-based estimate (*ca.* 0.15 Mya) of this population split derived from whole-genome sequences (Lescroart *et al.*, 2023). This is not surprising, since the TMRCA estimated from mitochondrial-based phylogenetic analyses is expected to track the split of mtDNA lineages in the ancestor that gave rise to descendant populations, prior to their actual divergence (Nei, 1987). Additional sampling of whole-genome sequences from multiple individuals of both regions will be required to further refine the estimate of the age and dynamics of the separation between these regional tigrina units.

The *L. guttulus* mitochondrial clade was reconstructed as the sister-group of this C. Am./Colombian tigrina clade. This node received low support (Figures 1, S1 and S2), likely due to the short length of the mitochondrial segment analyzed here, but is consistent with the mitogenome-based phylogeny reported by Lescroart et al. (2023). It is noteworthy that this mitochondrial topology is distinct from the most prevalent tree retrieved from the nuclear genome, which places the C. Am./Colombian group externally to an inner clade that includes *L. guttulus*, NE tigrina, *L. geoffroyi* and *L. guigna* (Trindade et al., 2021; Lescroart *et al.*, 2023). Such cyto-nuclear phylogenetic discordance has been shown to be common in the Felidae (e.g., Li et al., 2016, 2019), and part of the broader phylogenomic topological discordance that is prevalent in genus *Leopardus* (Lescroart *et al.*, 2023).

A remarkable finding was the phylogenetic position of Peruvian tigrinas, which had not been included in any of our previous studies. These samples formed a strongly supported clade (Figures 1, S1 and S2), which was more closely related to ocelots (*L. pardalis*), margays (*L. wiedii*) and the Andean cat (*L. jacobita*) than to any of the other tigrina units. This reconstruction, uniting this tigrina mtDNA clade with those belonging to the three species that comprise subgenus *Leopardus* (Lescroart et al., 2023), was strongly supported in both BI (1.0 posterior probability) and ML (95% bootstrap support) analyses. In addition, the node comprising the mtDNA clades belonging to subgenus *Oncifelis* (which includes all other tigrina units with autochthonous mtDNA, along with *L. geoffroyi* and *L. guigna*) also received high support (1.0 posterior probability and 99% ML bootstrap support). Taken together, these two strongly supported nodes robustly refute the monophyly of tigrina mtDNA clades, and show that these lineages are associated with two distinct subgenera within *Leopardus*. Interestingly, since the *L. colocola* complex comprises the third subgenus (*Lynchailurus*) within *Leopardus* (Lescroart *et al.*, 2023), the historical introgression of its mtDNA into NE tigrinas leads to a remarkable scenario in which present-day tigrina units harbor mitochondrial lineages stemming from all three subgenera within this genus. 

Another relevant result was that the divergence between the Peruvian tigrina clade and its sister lineage was dated at 2.09 Mya (95% HPD: 1.46 - 2.78 Mya), indicating an old evolutionary separation between this mtDNA lineage and those belonging to any other *Leopardus* unit. Taken together, the phylogenetic placement of this Peruvian tigrina mtDNA clade and depth of its evolutionary divergence represent a very surprising result, since these cats are morphologically similar to the other tigrinas (Nascimento and Feijó, 2017; de Oliveira et al., 2024), and were expected to be geographically, demographically, and genetically connected to the N. Andean (Colombian) population (now recognized as *L. pardinoides*). Instead, their mtDNA sequences grouped together strongly in a distinct clade, which was much more closely related to other *Leopardus* species, belonging to a different subgenus. Interestingly, despite the strong morphological similarity with specimens from Central America and northwestern South America, tigrinas from the Andes south of the Huancabamba depression (located in northwestern Peru) showed a slightly distinct spotting pattern, suggesting that they might comprise a different taxonomic unit (de Oliveira *et al.*, 2024), although the Huancabamba depression itself has so far not been retrieved as a historical barrier for ecological connectivity in this group (Bonilla-Sánchez et al., 2024). The genetic results presented here suggest that Peruvian tigrinas may (i) represent a distinct *Leopardus* taxon, either endemic to Peru or also occurring in other, yet-unsampled regions of South America; or (ii) bear an mtDNA lineage that has been introgressed in the past from a distinct (and potentially extinct) *Leopardus* species, similarly to what has happened between NE tigrinas and *L. braccatus* from the *L. colocola* complex (Trigo et al., 2013; Santos et al., 2018). Even in the latter case, the fact that these mtDNA lineages have so far only been found in Peru, and all eight sampled Peruvian tigrinas harbor these mitochondrial sequences, implies that this population is at least partially isolated from the other units (*i.e.*, at least the matrilineal gene flow between them is absent or very low). These hypotheses can be tested in the future using nuclear sequences and expanded sampling of tigrinas from other regions in South America.

From a biogeographic perspective, this finding is intriguing, since the ecological niche modeling conducted by Bonilla-Sánchez et al. (2024) did not detect a suitability break at the Huancabamba depression or any at other site that could explain a discontinuity between Colombian and Peruvian populations. That study did identify ecological niche differences between these two Andean regions, although these were subtler than could be expected given the depth of evolutionary divergence indicated by our mtDNA results (Bonilla-Sánchez *et al.*, 2024). Additional biogeographic and ecological data will be required to further investigate the spatial separation and potential adaptive differentiation between these units.

The inferred haplotype network (Figure 2) was congruent with the results of the phylogenetic analyses (Figure 1): each tigrina unit formed a well-defined haplogroup separated from each other by multiple mutational steps, indicating deep mitochondrial differentiation among these lineages. Although the position of the different outgroup sequences in this analysis was inconsistent (precluding a reliable inference of the *Leopardus* root), the mutational distances among the tigrina units were similar to, or higher than, the distances observed between other species in the genus (e.g., *L. pardalis vs. L. wiedii* or *L. geoffroyi vs. L. guigna*). In particular, the Peruvian unit was at least five mutational steps away from any other tigrina unit, the same distance that separated it from *L. pardalis*.

**Figure 2 -:**
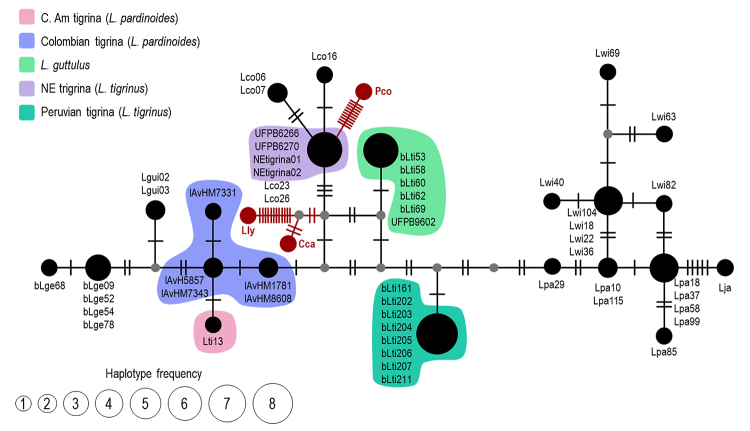
Haplotype network of mtDNA sequences from Neotropical felids of genus Leopardus, including all currently sampled geographic units of the *L. tigrinus* complex (color-coded as in Figure 1). Each circle represents a unique haplotype, with the circle diameter indicating haplotype frequency (see internal legend). Haplotype relationships are represented by connecting lines, on which crosslines indicate mutational steps. Small gray circles are median vectors that indicate inferred (extinct or unsampled) haplotypes. Due to the complete deletion of sites with any missing data, some closely related sequences shown in Figure 1 are collapsed into a single haplotype in this analysis. Outgroup species are represented by maroon circles (Cca: Caracal caracal; Lly: *Lynx lynx*; Pco: Puma concolor).

Overall, the present results demonstrate that mitochondrial polyphyly of the *L. tigrinus* complex is even more extreme than previously appreciated, and that additional taxonomic units may still be hidden within the ‘tigrina’ morphology. As previously suggested (Trindade et al., 2021), a plausible cause for this striking discrepancy between molecular phylogeny and morphological features is that the ‘tigrina’ phenotype may be ancestral (plesiomorphic) in the genus *Leopardus*. This would have led zoologists over the years to ‘lump’ distinct evolutionary units with a similar phenotype into the ‘*L. tigrinus*’ taxon, a problem that is only now being disentangled using molecular approaches and more refined morphological and ecological analyses. Hopefully the coming years will see a complete resolution of this problem enabled by the application of such improved approaches coupled with exhaustive geographic sampling across the whole *L. tigrinus* complex distribution.

## Supplementary Material

The following online material is available for this article:

Table S1 - Sample information.

Table S2 - Primers used to amplify the ND4-ND5 segment.

Figure S1 - Bayesian phylogeny of Leopardus mtDNA sequences, depicting posterior probabilities for all nodes.

Figure S2 - Maximum likelihood (ML) phylogeny of *Leopardus* mtDNA sequences.

## Data Availability

Novel sequences generated in this study have been deposited in GenBank (accession numbers PZ143437-PZ143486).
