# Supplementary material for: Strong evidence of mitochondrial polyphyly of the Leopardus tigrinus (Mammalia, Felidae) species complex revealed by expanded analyses of Andean populations
Source: Genet Mol Biol. 2026 Jul 17;49(2):e20250231. doi: 10.1590/1678-4685-GMB-2025-0231 (PMC13397909; doi:10.1590/1678-4685-GMB-2025-0231)
Supplement: Table S1 - [file 1415-4757-GMB-49-2-e20250231-s1.pdf]

Genetics and Molecular Biology
https://doi.org/10.1590/1678-4685-GMB-2025-0231

Supplementary Material to "Strong evidence of mitochondrial polyphyly of the Leopardus tigrinus (Mammalia, Felidae) species complex revealed by expanded analyses of Andean populations"

Table S1 - Sample information, including species identity, individual identification code, and provenance (country and locality, when available). For samples from Instituto Alexander von Humboldt (IAvH), sample codes correspond to their museum code. Samples with IDs beginning with bLge, bLti, NE_tigrina01, and NE_tigrina02 are part of the carnivoran biological sample collection of the PUCRS Laboratory of Genomics and Molecular Biology (Brazil). Samples IDs beginning with UFPB originate from the mammal collection of the Federal University of Paraíba (Brazil). Samples with IDs beginning with Lgui, Lco, Lti, Lpa and Lwi originate from the Laboratory of Genomic Diversity (NCI, NIH, USA) collection, currently housed at the Smithsonian Institution (Washington, DC, USA).

**Sample code**	**Species**	**Country***	**Locality**
bLge09	*L. geoffroyi*	AR	Córdoba
bLge52	*L. geoffroyi*	AR	Catamarca
bLge54	*L. geoffroyi*	UY	Montevideo
bLge68	*L. geoffroyi*	BO	Cochabamba Dept.
bLge78	*L. geoffroyi*	BR	Rio Grande do Sul, Taim
Lgui02	*L. guigna*	CH	captive origin (Zoo Nacional de Chile, Santiago)
Lgui03	*L. guigna*	CH	captive origin (Zoo Nacional de Chile, Santiago)
bLti53	*L. guttulus*	BR	Rio de Janeiro, Valença
bLti58	*L. guttulus*	BR	São Paulo, Americana
bLti60	*L. guttulus*	BR	São Paulo, Rafaro
bLti62	*L. guttulus*	BR	São Paulo, Campinas
bLti69	*L. guttulus*	BR	Rio Grande do Sul, Santa Cruz
Lco06	*L. pajeros*	AR	captive origin (Mendoza Zoo)
Lco07	*L. pajeros*	AR	captive origin (Cordoba Zoo)
Lco16	*L. garleppi*	PE	Pampa Galera
Lco23	*L. garleppi*	BO	La Paz
Lco26	*L. garleppi*	CH	Northern Chile
Lti13	*L. tigrinus*	CR	captive origin (Las Pumas Zoo)
Lpa58F	*L. pardalis*	BR	Paraná, Foz do Iguaçu
Lpa99F	*L. pardalis*	BO	Santa Cruz

Sample code	Species	Country*	Locality
Lpa85F	*L. pardalis*	VE	captive origin (Valencia aquarium)
Lpa10F	*L. pardalis*	BR	Amazonas, Barcelos
Lpa115F	*L. pardalis*	FG	French Guiana
Lpa29F	*L. pardalis*	GU	Petén
Lpa37F	*L. pardalis*	MX	Veracruz
Lpa18F	*L. pardalis*	PAN	captive origin (Summit Zoo)
Lwi63F	*L. wiedii*	BR	Pará, Belém
Lwi69F	*L. wiedii*	BR	Paraná, Foz do Iguaçu
Lwi82F	*L. wiedii*	BO	Beni
Lwi22F	*L. wiedii*	CR	Guanacaste
Lwi36	*L. wiedii*	GU	Petén
Lwi40F	*L. wiedii*	GU	captive origin (Auto Safari Chapin)
Lwi18F	*L. wiedii*	BR	Amazonas, Manaus
Lwi104F	*L. wiedii*	FG	French Guiana
bLti161	*L. tigrinus*	PE	captive origin (Parque de Las Leyendas, Lima)
bLti202	*L. tigrinus*	PE	Tarapoto
bLti203	*L. tigrinus*	PE	Tarapoto
bLti204	*L. tigrinus*	PE	captive origin (Zoo Patronato Parque de Las Legendas, Lima)
bLti205	*L. tigrinus*	PE	Tingo María
bLti206	*L. tigrinus*	PE	captive origin (Parque Huascar, Villa El Salvador, Lima)
bLti207	*L. tigrinus*	PE	captive origin (Parque Sinchi Roca, Comas, Lima)
bLti211	*L. tigrinus*	PE	Yurimaguas
UFPB9602	*L. guttulus*	BR	Paraíba
NE_tigrina01	*L. tigrinus*	BR	Cajazeiras, Paraíba
NE_tigrina02	*L. tigrinus*	BR	Brejo, Paraíba
IAvHM7331	*L. tigrinus*	CO	Quindío, Circasia, Reserva Forestal Bremen, Lat: 04°40'18,00"N, Long: 75°37'12,00"W, 1900m
IAvHM1781	*L. tigrinus*	CO	Boyacá, Coper, Región de Coper
IAvHM8608	*L. tigrinus*	CO	Boyacá, Villa de Leyva, SFF Iguaque, Vereda Capilla, cañón de mamaramos, 2800m
IAvH5857	*L. tigrinus*	CO	Nariño, PNN Volcán Galeras
IAvHM7343	*L. tigrinus*	CO	Caldas, Neira, Vereda La Cristalina, cuchilla El Guayabo, cuenca alta del río Tapias, Lat: 05°13'45,99"N, Long: 75°23'2,000"W, 3300m
UFPB6270	*L. tigrinus*	BR	Paraíba
UFPB6266	*L. tigrinus*	BR	Paraíba

*AR: Argentina; BO: Bolivia; BR: Brazil; CH: Chile; CO: Colombia; CR: Costa Rica; FG: French Guiana; GU: Guatemala; MX: Mexico; PAN: Panama; PE: Peru, UY: Uruguay; VE: Venezuela.
