# Supplementary material for: Strong evidence of mitochondrial polyphyly of the Leopardus tigrinus (Mammalia, Felidae) species complex revealed by expanded analyses of Andean populations
Source: Genet Mol Biol. 2026 Jul 17;49(2):e20250231. doi: 10.1590/1678-4685-GMB-2025-0231 (PMC13397909; doi:10.1590/1678-4685-GMB-2025-0231)
Supplement: Table S2 - [file 1415-4757-GMB-49-2-e20250231-s2.pdf]

Genetics and Molecular Biology
https://doi.org/10.1590/1678-4685-GMB-2025-0231

Supplementary Material to "Strong evidence of mitochondrial polyphyly of the Leopardus tigrinus (Mammalia, Felidae) species complex revealed by expanded analyses of Andean populations"

Table S2 - Primers used to amplify the ND4-ND5 segment. Several primers were used throughout the target ND4-ND5 fragments to allow amplification of the complete segment, particularly in skin samples.

Primer	Sequence (5' → 3')	Length	Tm
ND5-DLF1	TTGGTGCAACTCCAAATAAAAG	22 bp	57.1
ND5-DLR1	CGTAGTGGGGGTATAGGCTGT	21 bp	59.6
ND5-DLF2	CTGCCCATTATCATATCAAACACC	24 bp	55.9
ND5-DLR2	TTGAGATTACTGCTTCTTGTCCTG	24 bp	59.8
ND5-DLF3	AACCACAATCTCTTACGCCTTT	22 bp	59.3
ND5-DLR3	AGCTTAGCGATAGCTTGAGGGTTT	24 bp	63.3
