# Supplementary material for: Strong evidence of mitochondrial polyphyly of the Leopardus tigrinus (Mammalia, Felidae) species complex revealed by expanded analyses of Andean populations
Source: Genet Mol Biol. 2026 Jul 17;49(2):e20250231. doi: 10.1590/1678-4685-GMB-2025-0231 (PMC13397909; doi:10.1590/1678-4685-GMB-2025-0231)
Supplement: Figure S1 - [file 1415-4757-GMB-49-2-e20250231-s3.pdf]

Genetics and Molecular Biology

https://doi.org/10.1590/1678-4685-GMB-2025-0231

Supplementary Material to "Strong evidence of mitochondrial polyphyly of the Leopardus tigrinus (Mammalia, Felidae) species complex revealed by expanded analyses of Andean populations"

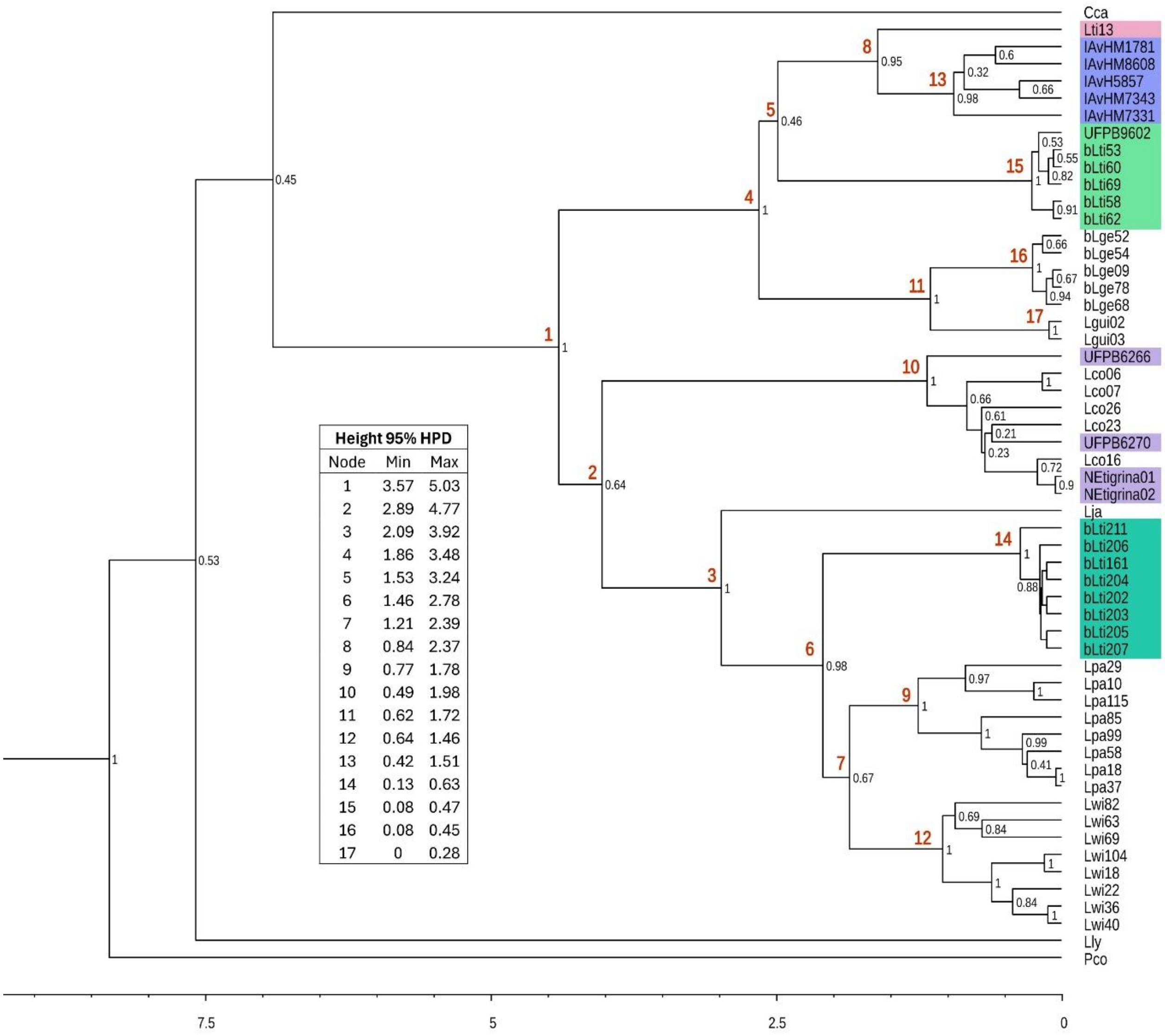

Height 95% HPD

Node	Min	Max
1	3.57	5.03
2	2.89	4.77
3	2.09	3.92
4	1.86	3.48
5	1.53	3.24
6	1.46	2.78
7	1.21	2.39
8	0.84	2.37
9	0.77	1.78
10	0.49	1.98
11	0.62	1.72
12	0.64	1.46
13	0.42	1.51
14	0.13	0.63
15	0.08	0.47
16	0.08	0.45
17	0	0.28

Figure S1 - Bayesian phylogeny of *Leopardus* mtDNA sequences, depicting posterior probabilities for all nodes. The main nodes are numbered, with the inset table depicting the corresponding height (divergence time in Mya) credibility interval (95% HPD interval) for each of them. Tigrina units are color-coded according to the map shown in Figure 1.
