# Supplementary material for: Strong evidence of mitochondrial polyphyly of the Leopardus tigrinus (Mammalia, Felidae) species complex revealed by expanded analyses of Andean populations
Source: Genet Mol Biol. 2026 Jul 17;49(2):e20250231. doi: 10.1590/1678-4685-GMB-2025-0231 (PMC13397909; doi:10.1590/1678-4685-GMB-2025-0231)
Supplement: Figure S2 - [file 1415-4757-GMB-49-2-e20250231-s4.pdf]

Genetics and Molecular Biology

https://doi.org/10.1590/1678-4685-GMB-2025-0231

Supplementary Material to “Strong evidence of mitochondrial polyphyly of the Leopardus tigrinus (Mammalia, Felidae) species complex revealed by expanded analyses of Andean populations”

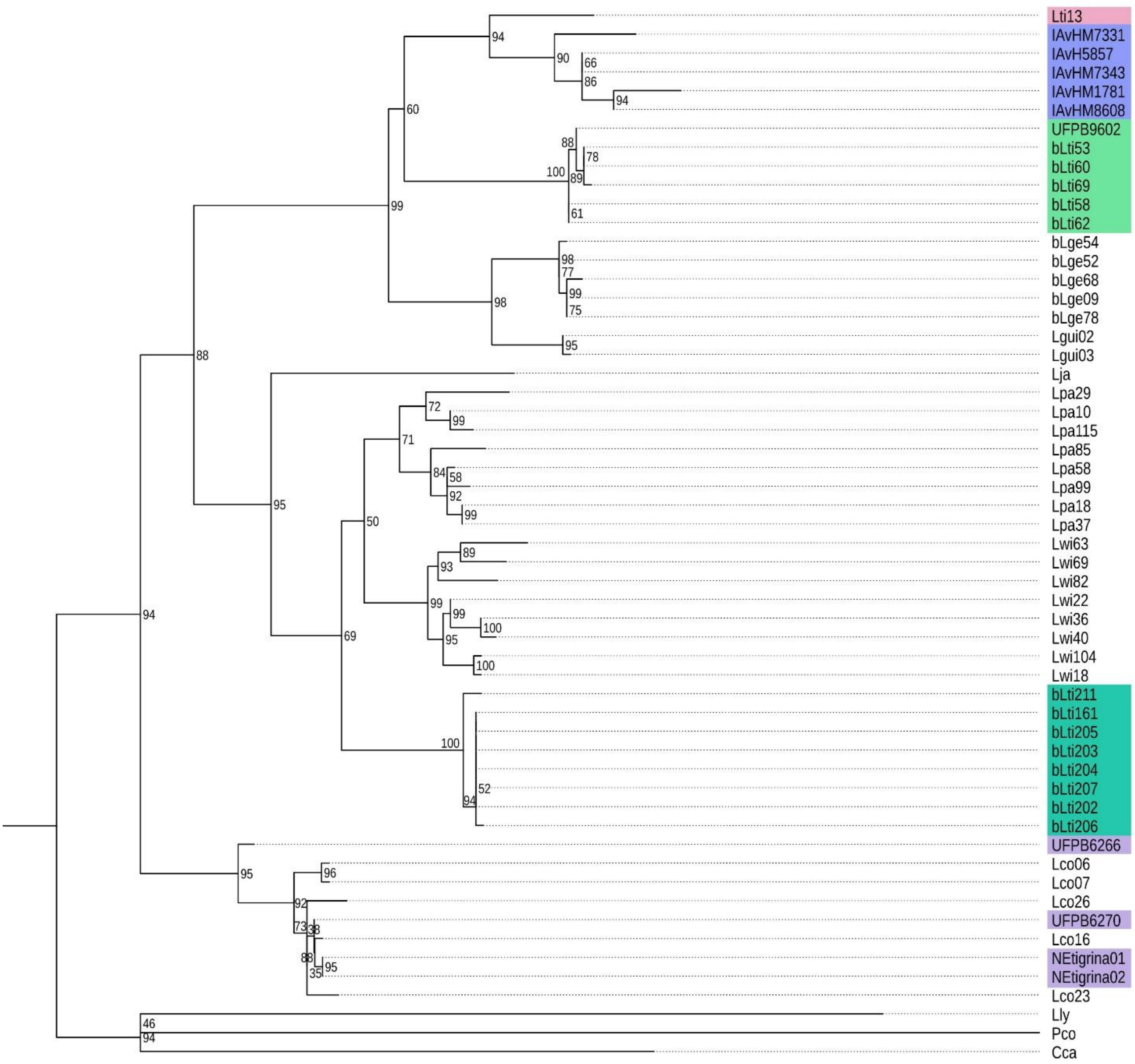

Figure S2 - Maximum likelihood (ML) phylogeny of *Leopardus* mtDNA sequences. Numbers next to nodes indicate their respective percent bootstrap support values. Tigrina units are color-coded according to the map shown in Figure 1.
